# Serum Cholesterol Differences Between Statin Users Who Take Dietary Supplements and Those Who Do Not: NHANES 2013–2018

**DOI:** 10.1016/j.cdnut.2022.100007

**Published:** 2022-12-23

**Authors:** Trisha S. MacDonald, Kathleen E. Davis, Wesley J. Tucker, Derek C. Miketinas

**Affiliations:** Texas Woman’s University – Houston Center, Houston, TX, USA

**Keywords:** NHANES, dietary supplements, cardiovascular disease, statins, cholesterol, HbA1c

## Abstract

**Background:**

Cardiovascular disease (CVD) is the leading cause of mortality in the United States and statins are the most commonly prescribed medication. It is important to understand the potential impact supplements may have when taken in combination with statins on serum lipid outcomes.

**Objectives:**

To evaluate the differences in the concentrations of cholesterol, triacylglycerol (TAG), and HbA1c between adults who use statins alone and those who combine statins and dietary supplements.

**Methods:**

A cross-sectional analysis using data from US adults aged ≥20 years who participated in the NHANES (2013–2018). The serum concentrations of lipids and the HbA1c levels were compared using independent sample t-tests. All analyses were adjusted for the complex survey design and used appropriate sample weights.

**Results:**

Of 16,327 participants included in this analysis, 13% reported the use of statins alone, and 8.8% used statins and dietary supplements. Statin users who used dietary supplements tended to be women (50.5%), aged 65.8 ± 0.4 years, and were more likely to be White (77.4%). Participants who used statins in combination with dietary supplements were less likely to have higher levels of total cholesterol (5.1% ± 1.4% vs. 15.6% ± 2.7%, *P* < 0.001), HbA1c (6.0% ± 0.1% vs. 6.3% ± 0.1%, *P* < 0.05), and HDL cholesterol (50 ± 1.3 vs. 47 ± 0.8 mg/dL, *P* < 0.05) than those who used statins alone. No significant differences were identified between the two groups for LDL cholesterol and TAG concentrations.

**Conclusions:**

Statin users who coingested dietary supplements were less likely to have high levels of total cholesterol and HbA1c and greater HDL levels than statin users who did not take dietary supplements. Dietary intake, lifestyle choices, and other confounders may have influenced the observed outcome differences for those who took dietary supplements with statins and those who did not.

## Introduction

Historically, drug interactions have been known for their potential adverse health outcomes [1]. These interactions arise when a drug’s mechanism of action is altered when coadministered with another pharmaceutical agent(s), food, beverage, or dietary supplement. Although only drug–drug interactions are included in pharmacovigilance systems to monitor the safety of drug products, drug–nutrient interactions can also alter drug effects that lead to clinically relevant outcomes [1]. Drug–nutrient interactions result from a physical, chemical, physiologic, or pathophysiologic relationship between a drug, nutrient, multiple nutrients, food in general, and nutritional status, and, thus, nutrition can improve or hinder a drug’s therapeutic effects to a varying degree [2]. Nutrient interactions are not included in these clinical assessments and remain underexplored, suggesting that the relevance of such interactions in public health has been undervalued and overlooked, leading to a lack of observational studies in this area of research [3]. The US population is aging; consequently, the prevalence of chronic diseases and the use of prescription medicines, nonprescription drugs, and dietary supplements have increased [4, 5]. It remains unclear how coingestion of dietary supplements and statins may impact lipid-lowering outcomes and the risk of CVD.

## Background

The use of dietary supplements in the United States (US) is on the rise [[6], [7], [8]]. The proportion of US citizens who take one dietary supplement daily has increased from 42% in 1988–1994 to over one-half of the population in 2003–2006 [7]. Dietary supplemental intake has continued to increase during 2017–2018, with 57.6% of adults aged ≥20 years reporting the use of supplements in the last 30 days [8]. The majority of consumers’ motivations for the use of dietary supplements have been to “improve” or “maintain” overall health [9]. Supplements such as multivitamins, vitamin D, niacin, red yeast rice, omega-3 FAs, and coenzyme Q10 (CoQ10) have been advocated for reducing the risk of select cardiovascular events. For instance, low vitamin D levels have been linked to the incidence of CVD [10]; CoQ10 supplementation has been shown to ameliorate symptoms of statin-induced myopathy [11]; omega-3 FA supplementation in the largest and longest randomized trial of daily usage has been shown to reduce the risk of heart attack, fatal heart attack, and total coronary heart disease (CHD) events by 28%, 50%, and 17%, respectively [12]; and red yeast rice, a natural source of monacolin K that is structurally identical to lovastatin, has gained popularity for its cholesterol-lowering action [13]. Thus, lipid levels and CVD outcomes may be impacted in those who use dietary supplements and statins concurrently.

CVD is a significant cause of morbidity and mortality and accounts for ∼659,000 deaths yearly among Americans [14]. The most prevalent of these conditions in the United States is CAD, which affects >18.2 million adults [14, 15]. CAD characteristically starts during childhood and progresses. Low-grade, chronic inflammation and plaque, a fatty substance formed from cellular waste products, calcium, fibrin, and, notably, cholesterol, are evident early on, and as the condition progresses into adulthood, plaque occludes and CHD results [15, 16]. Further, as cardiovascular events progress, so does therapy.

Statins are a pharmaceutical class of lipid-lowering drugs that reduce the levels of total cholesterol, LDL, and triacylglycerol (TAG) while simultaneously increasing the serum levels of HDL via selective inhibition of HMG-CoA reductase, the rate-limiting enzyme for endogenous synthesis of cholesterol in the liver [17]. Although the types and dosages of statins have varying capacities to lower LDL levels, reductions of 60% have been documented with rosuvastatin (Crestor) at a dosage of 40 mg/d [18]. By reducing the serum lipid levels closer to the normal range, statins reduce the risk of morbidity and mortality in patients with hypercholesterolemia, making them the first line of treatment for primary prevention of atherosclerotic CVD in patients with elevated LDL levels (>190 mg/dL), who are older (i.e., aged 40–75 years) and have comorbidities (e.g., diabetes mellitus), as recommended by the American College of Cardiology/AHA Task Force Clinical Practice Guidelines [19, 20]. Between 1999 and 2006, an estimated 29.0% of US citizens who had previously been diagnosed with high cholesterol levels took a statin medication [21]. Current evidence suggests that usage has increased, and the prevalence of the use of statins between 2007 and 2014 was reported to be 37.3% in those previously diagnosed with high cholesterol levels [21].

Drug evaluations for safety before and after market approval are primarily based on singular drug interactions or multiple medicinal substances [3]. Dietary supplements are formulated with various nutraceutical ingredients that vary in identity, potency, purity, and delivery matrix. They may contain a single ingredient or a combination of vitamins, minerals, botanicals, standardized plant actives, enzymes, amino acids, omega-3 FAs, soy derivatives, or probiotics [22]. Knowing that foods, nutrients, and drugs have complex relationships, it is challenging to identify the nature of those relationships in vivo. Drug efficacy while using supplemental nutrients can be impacted and may lead to unwanted side effects [23]. Further, evidence exists that there is lack of nutritional expertise and education in US medical schools, and physicians who report adverse drug reactions while using dietary supplements may be limited [3, 24].

Studies have provided evidence that select vitamins, minerals, and antioxidants may impact statin efficacy; however, there is little evidence regarding the concurrent use of statins and dietary supplements and its impact on serum cholesterol levels [[25], [26], [27], [28], [29], [30], [31]]. Given the increase in the concurrent use of statins and dietary supplements among US adults, improving our understanding of potential drug–nutrient interactions for this commonly prescribed and consumed class of drugs is important. Therefore, the primary purpose of this study was to investigate whether the cointake of statins and dietary supplements impacts the serum cholesterol, TAG, and HbA1c levels in adults aged ≥20 years compared with those in patients who use statins alone and supplements alone and those who take neither utilizing the NHANES, 2013–2018. The secondary purposes of this study were to investigate the prevalence of high levels of total cholesterol and LDL by sex, age, and ethnicity or race and estimate the average nutrient intake differences between statin users and those who cointake statins and dietary supplements.

## Methods

### Data sourcing and study population

This cross-sectional analysis included NHANES cycles from 2013 to 2018. The research methods of this study complied with the Declaration of Helsinki; furthermore, all survey participants signed informed consent documents, and their anonymity and confidentiality were retained per section 308(d) of the Public Health Service Act (42 U.S.C. 242m) [32, 33]. The original sample from the three combined cycles for persons of all ages included 29,400 participants. Of them, 16,327 were aged ≥20 years. Additionally, the FDA has requested the removal of the contraindication for the use of statin medications in pregnant women at a high risk of stroke or heart attack; therefore, pregnant women were included in this study [34].

### Demographic data

The demographic variables used in this analysis included age, gender, ethnicity or race, education level, income level, poverty income ratio, and marital status. Ethnicity or race was self-reported and initially categorized as Mexican American, other Hispanic, non-Hispanic White, non-Hispanic Black, non-Hispanic Asian, and other. A Hispanic group was later created by combining the Mexican American and other Hispanic groups [35]. Those in the “other” racial group were identified in the total sample; however, the results for this group were not shown separately when the prevalence of high cholesterol levels was ascertained [35]. The marital status was classified as married and not married. The income level was shown as four levels of household income groups dollars: missing or unknown, <$20,000, $20,000–$75,000, or ≥$75,000; it was also classified as the percentage of poverty income ratio: all individuals (those who did not report income), <130% of poverty, 130%–350% of poverty, and >350% of poverty [36]. Lastly, the education attainment level of the study participants was categorized as less than high school, high school diploma, or general equivalency diploma (GED), more than high school, or missing.

### Statin and dietary supplement user determination

NHANES participants report drug usage in the Prescription Medications subsection of the Sample Person Questionnaire Interview, which provides the therapeutic drug classes associated with each drug and ingredient. Statin users were defined for this analysis by identifying unique generic drug codes from the Multum Lexicon drug database. The statins that are commonly used include atorvastatin, simvastatin, lovastatin, pravastatin, fluvastatin, rosuvastatin, cerivastatin, and pitavastatin. Respondents were also interviewed about their use of dietary supplements. Those who reported supplementation within the past 30 days as per the DSD010 variable (i.e., Any Dietary Supplements Taken?) were classified as supplement users.

### Dietary data

Data from both 24-hour dietary recalls were utilized in addition to dietary information on the use of supplements. The nutrients of interest include energy, protein, carbohydrate, total sugars, cholesterol, dietary fiber, total fat, saturated FAs, monounsaturated FAs, polyunsaturated FAs, and micronutrients because they may affect serum cholesterol levels, weight status, the risk of CVD, and the efficacy of statins [26, 37, 38]. The dietary intake for the two assessment periods was combined and averaged with and without the contribution of dietary supplements for each of the four groups to ascertain the differences in dietary intake among statin users, those who cointake statins and dietary supplements, those who use supplements alone, and those who use neither. The Healthy Eating Index (HEI) 2015 scores were also calculated to assess the differences in diet quality among the groups for total scores and all 13 individual components.

### Anthropometric data

For this analysis, height, weight, and waist circumference were of interest because obesity is an associated risk factor for hypertension, dyslipidemia, diabetes, and elevated levels of fibrinogen and markers for inflammation, all of which increase the risk of CVD events such as stroke, myocardial infarction, and pulmonary embolism [39, 40]. BMI was calculated in kg/m^2^. BMI was classified into the following categories: underweight (BMI < 18.5), normal weight (18.5 ≤ BMI < 25), overweight (25 ≤ BMI < 30), or obese (BMI ≥ 30) [41].

### Serum measurements

Trained staff collected blood and specimens at a mobile examination center. Data on the serum levels of total cholesterol, HDL, LDL, and TAG as well as HbA1c levels were utilized for the analysis in this study. High levels of total cholesterol were defined as ≥240 mg/dL; high TAG levels were defined as ≥200 mg/dL; high LDL cholesterol levels were defined as ≥160 mg/dL; elevated HbA1c levels were defined as >5.7%, whereas low HDL cholesterol levels were defined as <40 mg/dL [42].

## Smoking

Participants who answered “No” to “Smoked at least 100 cigarettes in life” were classified as “Never smoker.” Those who answered “Yes” and then answered “Not at all” to “Do you now smoke cigarettes” were classified as “Past smoker.” Those who answered “Yes” to “Smoked at least 100 cigarettes in life” and “Every day” or “Some days” to “Do you now smoke cigarettes” were classified as “Current smoker.”

### Statistical analysis

Continuous variables are presented as means ± SEM and categorical variables as percentages. Sample weights were adjusted to account for the three survey cycles included in this analysis, as recommended by the NHANES Analytical Guidelines [43]. All estimates were age adjusted by the projected 2010 US population using age groups 20–39, 40–59, and ≥60 years [44]. The demographic variables included age, gender, ethnicity or race, education level, income, poverty income ratio, and marital status. Ethnicity was classified as Mexican American, other Hispanic, non-Hispanic White, non-Hispanic Black, non-Hispanic Asian, and other. However, when the prevalence of high cholesterol levels was estimated, the Mexican American and other Hispanic categories were merged because Mexican Americans comprise a limited portion of the unweighted sample size and may not have met the analytical criteria outlined in the NHANES Analytic Guidelines required for analysis if analyzed separately.

Descriptive statistics were generated using correct sampling weights and accounted for the survey design. Estimates were generated across the four groups of interest: those who cointake statins and supplements, those who use statins and no supplements, those who take supplements and no statins, and those who use neither. Proc surveyreg was used to generate age-adjusted estimates using the 2010 census for the serum levels of cholesterol, LDL, HDL, and TAG as well as HbA1c levels. We used proc surveyreg to test whether the outcomes of interest varied across the four groups. The covariates of interest included age, gender, ethnicity or race, education level, income level, marital status, BMI, waist circumference, and smoking status. In this study, all statistical analyses were performed utilizing SAS, version 9.4 (SAS Institute Inc.). A *P* value of <0.05 was considered statistically significant.

## Results

**Figure 1:**
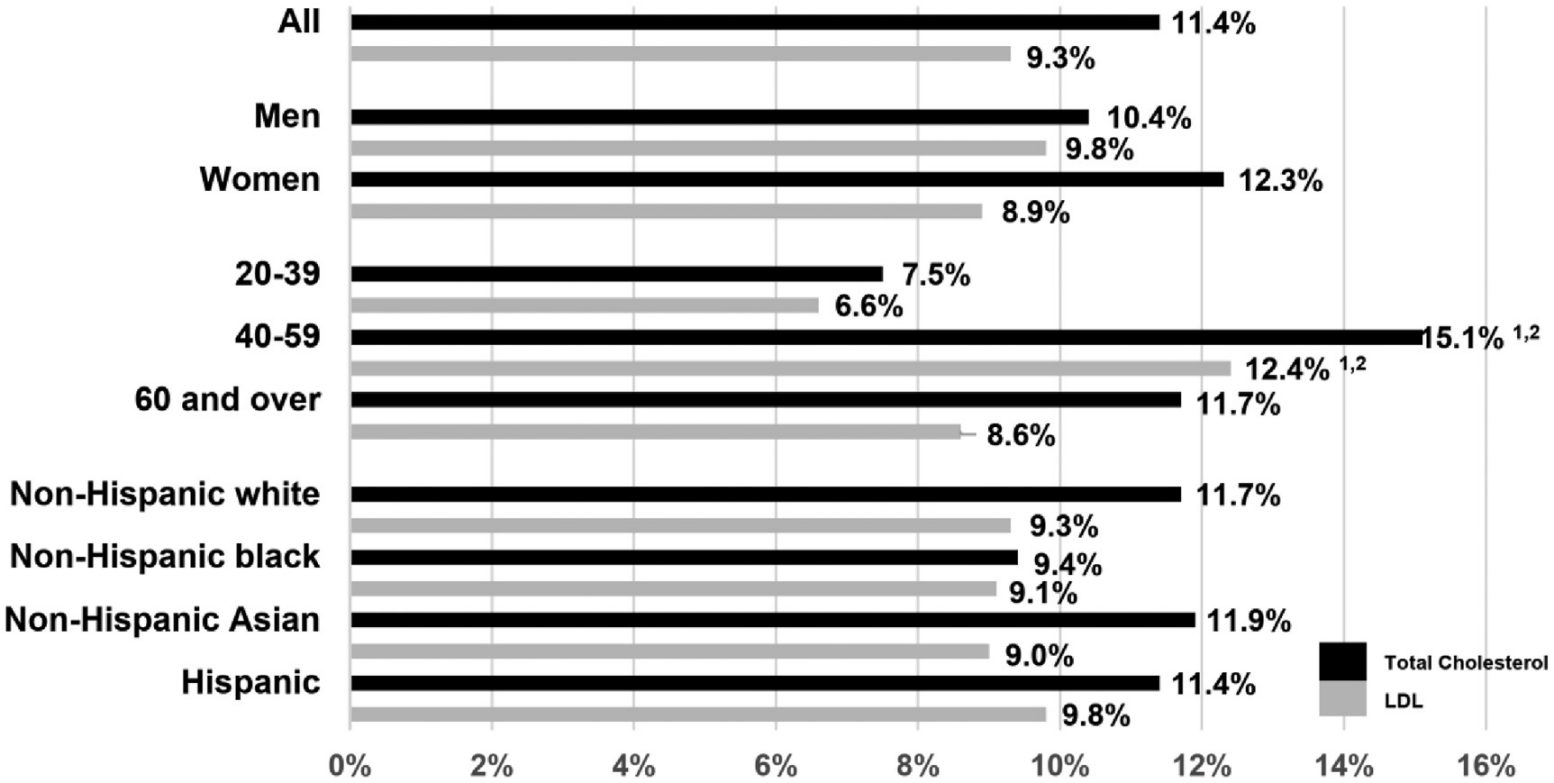
Prevalence of high levels of total and LDL cholesterol among adults aged ≥20 years by sex, age, and race and Hispanic origin: United States, 2013–2018.

From 2013 to 2018, it was found that 11.4% and 9.3% of adult participants who were aged ≥20 years had elevated levels of total cholesterol (≥240 mg/dL) and LDL (≥160 mg/dL), respectively. Adult participants aged 40–59 years had a higher prevalence of high levels of cholesterol and LDL than those aged 20–39 and ≥60 years (Figure 1). Additionally, women (12.3%) had an even greater percentage of cholesterol levels >240 mg/dL than men (10.4%); however, this finding was not significant. In contrast, a greater percentage of men had serum LDL levels >160 mg/dL than that of women. No significant differences among the ethnic groups were found to exist for the prevalence of high levels of cholesterol or LDL.

Of the 16,327 participants in this study, 2142 were statin users (13.0%). The commonly used statins among the study groups included atorvastatin (35.0%) and simvastatin (32.5%) in addition to pravastatin (14.8%), rosuvastatin (11.4%), and lovastatin (6.3%). Of these statin users, 67% said “Yes,” they had taken supplements in the last 30 days. Of those who used both statins and dietary supplements (*n* = 1436), 50.5% were women, with an average age of 65.8 ± 0.4 years; they were more likely to be White (77.4%) and married (63.3%) and had an education level of high school diploma (GED) or greater (77.7%). Those who used statins and supplements had an average BMI of 30.7 ± 0.3; 46.1% were classified as obese, with a waist circumference of 106.1 ± 0.6 cm (Table 1).

**Table 1:** Participant characteristics of adults aged ≥20 years by statin and supplement use status: NHANES 2013–2018

Statin and supplement use categories					
	Total	Statins + supplements	Statins alone	Supplements alone	Neither
	*n* = 16,327	*n* = 1436	*n* = 706	*n* = 7220	*n* = 6965
Age, y	47.9 ± 0.3	65.8 ± 0.4	61.3 ± 0.6	49.7 ± 0.4	40.9 ± 0.2
Gender, %					
Male	48.1	49.5	60.1	40.1	54.6
Female	51.9	50.5	39.9	59.9	45.4
Race, %					
Mexican American	8.9	4.1	6.0	7.0	12.5
Other Hispanic	6.3	2.4	4.5	5.7	8.0
Non-Hispanic White	63.9	77.4	71.4	68.4	55.3
Non-Hispanic Black	11.4	8.4	12.6	9.1	14.6
Non-Hispanic Asian	5.7	4.6	2.9	6.0	5.8
Other	3.7	3.1	2.6	3.8	3.8
Education^4^, %					
Missing or unknown	0.1	0.1	0.1	0.1	0.2
Less than high school	13.7	22.2	34.0	16.0	26.5
High school diploma or GED^2^	55.1	52.4	48.7	52.8	54.9
More than high school	31.1	25.3	17.1	31.3	18.5
Income, %					
Missing or unknown	7.7	6.4	5.2	6.0	6.4
< $20,000	20.8	31.2	38.5	23.9	32.1
$20,000–$74,999	36.8	38.9	36.1	40.5	41.4
≥$75,000	34.7	23.5	20.1	29.6	20.2
Poverty income ratio^5^, %					
All individuals	8.5	9.3	8.6	10.3	10.8
<130% of poverty	20.2	24.6	35.0	22.5	35.1
130%–350% of poverty	32.8	35.2	32.9	34.7	34.2
>350% of poverty	38.6	30.9	23.5	32.5	19.9
Marital status, %					
Married	54.3	63.3	63.2	57.2	48.1
Unmarried	45.7	36.7	36.8	42.8	51.9
BMI^3^ (kg/m^2^)^1^	29.5 ± 0.1	30.7 ± 0.3	31.0 ± 0.4	29.0 ± 0.2	29.7 ± 0.2
BMI, %					
Underweight	2.6	2.0	2.4	2.4	2.9
Normal weight	26.2	17.0	16.3	28.5	26.3
Overweight	31.5	34.9	31.4	32.0	30.3
Obese	39.7	46.1	49.9	37.1	40.5
Waist circumference, cm^1^	100.3 ± 0.4	106.1 ± 0.6	107.7 ± 0.8	99.0 ± 0.4	100.0 ± 0.4
Smoking status^4^, %					
Never	56.9	48.5	44.5	60.2	55.8
Former	24.6	38.7	36.7	25.8	19.0
Current	18.6	12.9	18.8	14.0	25.1

1 Values are mean ± SEM unless otherwise indicated.

2 GED, general equivalency diploma.

3 BMI, body mass index. BMI (calculated as kg/m^2^) categories—normal weight, 18.5 ≤ BMI < 25; overweight, 25 ≤ BMI < 30; obese, BMI ≥ 30; and underweight, BMI < 18.5.

4 Education and smoking status: missing values were excluded because of the small sample size.

5 Poverty income ratio: “all individuals” includes individuals whose income was not reported.

The age-adjusted mean estimates for the levels of total cholesterol, LDL, HDL, and TAG as well as HbA1c levels of the study sample were 191 mg/dL, 112 mg/dL, 54 mg/dL, 117 mg/dL, and 5.7%, respectively (Table 2). Those who took supplements alone had the lowest levels of TAG and HbA1c; they also had the highest HDL levels among the four groups. Those who took statins and supplements concurrently had the lowest levels of total cholesterol and LDL across the four groups. When the use of statins and dietary supplements was compared with that of statins alone, lower mean values of the total levels of cholesterol, LDL, and TAG as well as elevated HbA1c levels (≥5.7%) were observed but were not statistically significant. Those who reported the cointake of statins and supplements had a lower prevalence of high cholesterol levels (*P* < 0.001), lower HbA1c levels (*P* < 0.05), and greater HDL levels (*P* < 0.05) than those who reported the use of statins alone.

**Table 2:** Serum lipid concentrations and hemoglobin A1c for the total sample and across statin and supplement use in adults who participated in the NHANES 2013–2018

Statin and supplement use categories					
	Total	Statins + supplements	Statins alone	Supplements alone	Neither
Total cholesterol, mg/dL	191 ± 0.8	178 ± 2.9	187 ± 4.2	195 ± 1.0	192 ± 0.9
High cholesterol levels, %	11.4 ± 0.42	5.1 ± 1.40^1^	15.6 ± 2.73	12.8 ± 0.68	12.3 ± 0.63
Triacylglycerols, mg/dL	117 ± 1.9	125 ± 8.6	125 ± 10.8	114 ± 2.5	117 ± 2.7
High triacylglycerol levels, %	11.6 ± 0.54	12.9 ± 3.00	11.5 ± 2.69	10.4 ± 0.77	12.0 ± 1.06
LDL cholesterol, mg/dL	112 ± 0.7	100 ± 2.5	101 ± 4.2	117 ± 1.2	118 ± 1.2
High LDL cholesterol levels, %	9.3 ± 0.68	2.6 ± 1.50	5.2 ± 2.19	10.6 ± 0.90	12.5 ± 1.17
HDL cholesterol, mg/dL	54 ± 0.3	50 ± 1.3^2^	47 ± 0.8	57 ± 0.4	52 ± 1.5
Low HDL cholesterol levels, %	18.0 ± 0.60	23.9 ± 4.84	33.4 ± 4.21	14.3 ± 0.58	20.7 ± 1.09
HbA1c, %	5.7% ± 0.1	6.0% ± 0.1^2^	6.3 ± 0.1	5.6 ± 0.0	5.7 ± 0.0
Elevated HbA1c levels, %	26.5 ± 4.70	49.5 ± 6.81	51.8 ± 4.25	23.4 ± 7.29	25.6 ± 8.20

Notes: High total cholesterol level is defined as ≥240 mg/dL. High triacylglyceride level is defined as ≥200 mg/dL. High LDL cholesterol level is defined as ≥160 mg/dL. Low HDL cholesterol level is defined as <40 mg/dL. Elevated hemoglobin A1c level is defined as >5.7%. All estimates are age-adjusted by the projected 2010 US population using age groups 20–39, 40–59, and ≥60 years.

1 Significantly different from statins alone (*P* < 0.001).

2 Significantly different from statins alone (*P* < 0.05).

The mean dietary intake was assessed for those who used statins alone and those who used statins in combination with dietary supplements. Compared with those who used statins alone, those who used statins and supplements had similar nutrient intake from food sources alone; however, significant differences did exist for caloric (*P* = 0.0447), vitamin C (*P* = 0.0099), niacin (*P* = 0.0144), copper (*P* = 0.0406), and selenium (*P* = 0.0485) intakes (Table 3). When nutrient intake was assessed for nutrient intake from both foods and supplements, significant differences between the 2 groups were observed—vitamin C (*P* < 0.0001), vitamin D (*P* < 0.0001), vitamin B_1_ (*P* = 0.0069), vitamin B_2_ (*P* < 0.0001), niacin (*P* < 0.0001), folate (*P* < 0.0001), vitamin B_6_ (*P* < 0.0001), vitamin B_12_ (*P* < 0.0001), calcium (*P* < 0.0001), magnesium (*P* < 0.0001), iron (*P* < 0.0001), zinc (*P* < 0.0001), copper (*P* < 0.0001), and selenium (*P* = 0.0016) (Table 3).

**Table 3:** Mean nutrient intakes for adults aged ≥20 years from foods alone and foods and supplements combined: NHANES 2013–2018

	Foods alone			Foods and supplements		
	Statins + supplements	Statins alone	*P* value	Statins + supplements	Statins alone	*P* value
Energy, kcal/d	1917 ± 26	2036 ± 50	0.0447^1^	1925 ± 26	2037 ± 50	0.0600
Protein, g/d	76.5 ± 1.10	80.9 ± 2.23	0.1113	76.7 ± 1.1	80.9 ± 2.2	0.1245
Carbohydrate, g/d	223.6 ± 3.39	232.85 ± 5.7	0.1729	224.3 ± 3.4	232.9 ± 5.7	0.2056
Total sugars, g/d	95.5 ± 2.00	97.3 ± 3.0	0.6432	95.8 ± 2.0	97.3 ± 3.0	0.6906
Dietary fiber, g/d	17.0 ± 0.3	16.5 ± 0.7	0.5280	17.1 ± 0.3	16.6 ± 0.6	0.4215
Cholesterol, mg/d	278.2 ± 5.7	301.9 ± 12.2	0.0817	280.9 ± 5.7	302.0 ± 12.2	0.1215
Total fat, g/d	77.2 ± 1.3	82.9 ± 2.9	0.1016	77.7 ± 1.3	82.9 ± 2.9	0.1336
Total saturated FAs, g/d	24.9 ± 0.5	26.8 ± 1.0	0.1139	25.0 ± 0.5	26.8 ± 1.0	0.1222
Total monounsaturated FAs, g/d	27.1 ± 0.5	29.2 ± 1.1	0.0871	27.1 ± 0.5	29.2 ± 1.1	0.0889
Total polyunsaturated FAs, g/d	18.1 ± 0.3	19.2 ± 0.7	0.1988	18.2 ± 0.3	19.2 ± 0.7	0.2360
Vitamin C, mg/d	82.5 ± 3.2	70.2 ± 3.8	0.0099^1^	226.2 ± 12.6	76.1 ± 4.8	<0.0001^1^
Vitamin D, μg/d	4.7 ± 0.1	4.9 ± 0.3	0.5427	41.4 ± 3.4	7.1 ± 0.6	<0.0001^1^
Vitamin B_1_, mg/d	1.5 ± 0.0	1.6 ± 0.1	0.1331	10.6 ± 3.1	1.9 ± 0.2	0.0069^1^
Vitamin B_2_, mg/d	2.1 ± 0.0	2.2 ± 0.1	0.3311	5.2 ± 0.4	2.5 ± 0.2	<0.0001^1^
Niacin, mg/d	23.7 ± 0.3	25.6 ± 0.7	0.0144^1^	42.6 ± 1.9	27.8 ± 1.3	<0.0001^1^
Folate, μg DFE/d	507.0 ± 13.2	482.1 ± 14.7	0.2330	947.6 ± 42.5	509.3 ± 16.6	<0.0001^1^
Vitamin B_6_, mg/d	2.0 ± 0.1	2.1 ± 0.1	0.2449	7.1 ± 0.8	2.6 ± 0.2	<0.0001^1^
Vitamin B_12_, μg/d	5.2 ± 0.3	4.9 ± 0.4	0.6149	166.4 ± 26.5	12.9 ± 5.1	<0.0001^1^
Vitamin K, μg/d	111.9 ± 3.3	111.0 ± 6.9	0.9072	127.00 ± 3.7	111.8 ± 7.0	0.0660
Choline, mg/d	324.6 ± 4.8	345.8 ± 11.8	0.0864	326.3 ± 4.8	345.9 ± 11.9	0.1114
Calcium, mg/d	900.0 ± 18.3	895.5 ± 27.3	0.8911	1221.4 ± 24.6	953.5 ± 32.2	<0.0001^1^
Magnesium, mg/d	291.8 ± 5.1	289.0 ± 7.7	0.7414	343.9 ± 7.0	294.4 ± 7.8	<0.0001^1^
Phosphorus, mg/d	1306.0 ± 20.9	1338.8 ± 36.9	0.4629	1320.1 ± 20.8	1339.3 ± 36.9	0.6689
Potassium, mg/d	2654.4 ± 32.6	2686.6 ± 77.5	0.7096	2690.9 ± 33.3	2692.9 ± 77.8	0.9819
Iron, mg/d	14.2 ± 0.3	14.1 ± 0.5	0.8627	21.3 ± 0.7	14.7 ± 0.5	<0.0001^1^
Zinc, mg/d	10.7 ± 0.2	11.0 ± 0.4	0.6336	18.8 ± 0.4	11.8 ± 0.5	<0.0001^1^
Copper, mg/d	1.3 ± 0.1	1.2 ± 0.0	0.0406^1^	1.8 ± 0.1	1.2 ± 0.0	<0.0001^1^
Selenium, μg/d	107.6 ± 1.8	116.2 ± 3.8	0.0485^1^	134.4 ± 2.5	118.4 ± 3.7	0.0016^1^

Notes: Dietary mean intake was calculated using a mean of 2 24-Hour Dietary Recal1s (if present). Values are mean ± SEM unless otherwise indicated.

DFE, Dietary Folate Equivalent.

1 A *P* value of <0.05 was considered statistically significant.

Those who co-used statins and supplements had a significantly higher total HEI score than those who used statins alone (51.2; 95% CI: 50.3, 52.0 vs. 47.8; 95% CI: 46.7, 48.9, respectively; *P* < 0.0001) (Table 4). For the HEI individual category component assessment scores, those who used supplements and statins had higher intakes of total fruits (*P* < 0.0001), whole fruits (*P* < 0.0001), total vegetables (*P* = 0.0095), greens and beans (*P* = 0.0227), whole grains (*P* = 0.0019), total dairy (*P* = 0.0057), total protein foods (*P* = 0.0013), and seafood and plant proteins (*P* < 0.0001) (Table 4).

**Table 4:** Healthy Eating Index-2015 scores for adults aged ≥20 years who consumed statins and supplements combined and those who did not: NHANES 2013–2018^1^

Component	Range	Statins + supplement use Mean (95% CI)	MS^2^, %	Statins use alone Mean (95% CI)	MS^2^, %	*P* value
Total fruits	0–5	2.0 (1.8, 2.2)	40	1.4 (1.3, 1.5)	28	<0.0001
Whole fruits	0–5	2.2 (2.0, 2.4)	44	1.6 (1.4, 1.7)	32	<0.0001
Total vegetables	0–5	2.3 (2.2, 2.4)	46	2.0 (1.8, 2.1)	40	0.0095
Greens and beans	0–5	1.1 (1.0, 1.2)	22	0.9 (0.7, 1.1)	18	0.0227
Whole grains	0–10	2.5 (2.2, 2.7)	50	1.8 (1.6, 2.1)	36	0.0019
Total dairy	0–10	3.6 (3.4, 3.9)	36	3.2 (2.8, 3.5)	32	0.0057
Total protein foods	0–5	3.2 (3.1, 3.3)	64	2.9 (2.8, 3.1)	58	0.0013
Seafood and plant proteins	0–5	2.0 (1.9, 2.2)	40	1.6 (1.4, 1.7)	32	<0.0001
FAs	0–10	4.5 (4.3, 4.7)	45	4.5 (4.2, 4.9)	45	0.8551
Refined grains	0–10	8.3 (8.1, 8.4)	83	8.4 (8.1, 8.7)	84	0.5343
Sodium	0–10	4.2 (3.9, 4.6)	42	4.4 (3.9, 4.8)	44	0.7331
Added sugars	0–10	8.5 (8.3, 8.7)	85	8.6 (8.4, 8.9)	86	0.3261
Saturated fats	0–10	6.7 (6.5, 6.9)	67	6.6 (6.2, 7.04)	66	0.8592
Total score	0–100	51.2 (50.3, 52.0)	51	47.8 (46.7, 48.9)	48	<0.0001

MS, maximum score.

^1^*n* = 1328 statins + supplement use; *n* = 633 statin use alone. The HEI scores were calculated with the use of the population ratio method.

^2^ Mean values as a percentage of the maximum component score.

1 Significantly different from adults aged 20–39 years.

2 Significantly different from adults aged ≥60 years.

## Discussion

Statins are a class of pharmaceutical drugs with a remarkable track record for reducing the levels of total cholesterol, LDL, and TAG and increasing HDL levels by selectively inhibiting HMG-CoA reductase, reducing the risk of morbidity and mortality in patients with CVD [17, 19]. As a result, the use of statins increased by 79.8% from 2002–2003 to 2012–2013 in US adults aged ≥40 years [45]. This study showed that over half of the participants used dietary supplements within the last 30 days. Of those taking statins, 67% were also taking dietary supplements. Those who did not take statins were 1.95 times more likely to take supplements than those who did take statins (OR: 1.95; 95% CI: 1.61, 2.36) (data not shown). The concurrent use of statins and dietary supplements may increase the potential for drug–nutrient interactions. However, the exploration of the efficacy of statins in serum cholesterol management during concurrent use of dietary supplements is limited [[25], [26], [27], [28], [29], [30], [31], 46, 47]. To the best of our knowledge, this is the first cross-sectional study of its kind that assessed the mean differences in the serum levels of lipids between those who take statin drugs and dietary supplements and those who take statins alone. We found that the NHANES participants who used statins in combination with dietary supplements were less likely to have high total cholesterol levels (5.1% ± 1.4% vs. 15.6% ± 2.7%, *P <* 0.001), had lower HbA1c levels (6.0% ± 0.1% vs. 6.3% ± 0.1%, *P* < 0.05), and were more likely to have higher HDL levels (50 ± 1.3 vs. 47 ± 0.8 mg/dL, *P <* 0.05) than those who used statins alone. Those who used statins alone had lower levels of total cholesterol, LDL, and HDL than those who used supplements alone or neither; however, the difference in the total cholesterol level was much more pronounced when the use of statins was combined with that of dietary supplements (178 ± 2.9 vs. 187 ± 4.2 mg/dL). However, this finding was not statistically significant. The HDL levels in those who used statins and supplements were significantly greater than in those who used statins alone, with a mean difference of 3 mg/dL (*P* < 0.05). Lastly, statin therapy aims to reduce the levels of LDL and TAG. Although not statistically significant, the participants who used statins in combination with dietary supplements were less likely to have high LDL levels (i.e., ≥160 mg/dL) than those who used statins alone (2.6% ± 1.5% vs. 5.2% ± 2.2%, respectively). No differences were observed for TAG.

The role of dietary supplements and their impact on health and wellness have been intensely studied; however, high-quality, long-term randomized studies are lacking, and the research is confounded by healthy lifestyle choices and eating practices that typically adjoin with the use of supplements. Based on the 2021 NCHS Data Brief for Dietary Supplement Use, the most common dietary supplements used among adults are as follows: *1*) multivitamin–mineral formulas, *2*) vitamin D, and *3*) omega-FAs for all ages [8]. Given the high intake of some nutrients, such as vitamins C, D, B_1_, B_2_, and niacin, among those who use statins and supplements, is it possible that a synergetic relationship between the use of statins and that of dietary supplements exists and impacts lipid profile outcomes? Fiber has been well documented for its function in improving the serum levels of lipids and improving the efficacy of statins [37]. Although the soluble portion of fiber slows the absorption rate of cholesterol from the diet, it may also prolong the cholesterol-lowering effects of statin drugs [37]. However, this study showed no difference in fiber intake between the groups. Vitamin D is of interest because adequate intake from food and sun sources alone can be challenging. Vitamin D in all its metabolite forms, except 1,25-dihydroxyvitamin D_3_, inhibits HMG-CoA reductase activity, reducing the biosynthesis of hepatic cholesterol and circulating cholesterol levels in vitro [48]. The effect of vitamin D on the pharmacodynamics and pharmacokinetics of statins has been tested in clinical trials [26, 31, 46, 47, 49]. However, the results were not consistent [46, 47]. CoQ10 is a fat-soluble organic molecule that works in the electron transport chain, converting food to energy, and its deficiency is common in myocardial biopsies from various CVD events [50]. CoQ10 is dependent on endogenous synthesis of cholesterol in the liver, and the use of statins hinders its production. The consequences of the use of statins on CoQ10 levels and the effect of the degree of mitochondrial function may be susceptible to genetic predisposition [51]. However, whether CoQ10 supplementation is beneficial during the use of statins is still uncertain. Lastly, omega-3 fish oil supplementation has shown promising benefits in statin users. For example, the Reduction of Cardiovascular Events with Icosapent Ethyl–Intervention Trial showed that 2 g of the omega-3 FA eicosapentaenoic acid twice daily reduced elevated TAG levels (135–499 mg/dL), cardiovascular events, and CVD-related death in patients with high TAG levels who were on statin therapy but with controlled LDL levels [52]. This was further acknowledged in large-scale epidemiologic studies that acknowledged that the consumption of omega-3 FAs, either as fish or supplements, may benefit those who are at the risk of CHD [53]. The Dietary Guidelines for Americans from 2015 to 2020 also recommends omega-3 FAs, 450–500 mg/d for general health and 1 g/d for those with CHD but with the sourcing preference being fatty fish [54]. This study found no difference in the TAG levels between those who used statins alone and those who used statins in combination with dietary supplements.

The strengths of this study include using multiple cycles of the NHANES, which provides reliable estimates from a nationally representative sample of the civilian, non-institutionalized US population, especially because there is oversampling of older individuals and minority populations are included in the NHANES. Additionally, the findings of this analysis are representative of adults aged ≥20 years.

There are limitations to this analysis. The differences observed in the high levels of total cholesterol, HbA1c, and HDL between those who used statins and supplement and those who used statins alone may have been confounded by extraneous variables, including type and dosage level of supplements, type and dosage level of statins, or additional confounding factors such as adherence to healthy eating patterns. Although HEI scoring is of interest, it cannot be used as a covariate in this model because it would introduce measurement errors. Further, this study cannot attribute these differences to a single nutrient. The categorical variable used to ascertain the use of dietary supplements is broad because it includes all preparations, single and multinutrient options, vitamins, minerals, herbs, omegas, probiotics, and others. The dietary supplement products consumed by the NHANES participants may have had a wide range of dosage levels. Additionally, this analysis only identified the use of statins as a whole and did not analyze the data for significance by the types of statins, multiple use, length of use, or dosage level of the individual statins. It is important to note that statins vary in dosage, pharmacokinetics, pharmacodynamics, intensity level, solubility, and biotransformation. Although the approach used in this study provides an unbiased estimate of the usual mean intake, the NCI method is a more appropriate methodology for estimating the distributions of usual intake, which can, in turn, be used to fit regression models to identify the relationship between nutrient intake and CVD outcomes.

This study is broad, including supplement users as a group, when they may have been heterogeneous in their use of CVD-protective, -neutral, or -negative supplements. However, given the limitations of the NHANES question about the use of supplements, this is an unavoidable limitation of using this data set. Still, monitoring the safety of drug products and detecting the impact of drug outcomes with the concurrent use of supplements are important for patient health outcomes. Although causation cannot be inferred from this cross-sectional study, these findings highlight the need to explore drug–nutrient interactions for commonly prescribed drugs, particularly those for statins.

In conclusion, this study suggests that US adults aged ≥20 years who use statins and concurrently take dietary supplements have a lower prevalence of high cholesterol levels, lower HbA1c levels, and greater HDL levels than those who use statins alone. These findings highlight the potential for a synergistic effect between statins and supplements. However, there is a possibility that confounders, such as healthy eating factors, or social determinants of health, such as economic stability, and other variables, such as physical activity, were present and not accounted for in this study. Additional studies are needed to identify the specific vitamin, mineral, antioxidant, combination formula, or dosage range responsible for improving serum cholesterol levels. In particular, subsequent analyses should further examine the supplemental intake of a single ingredient or even combination dietary supplement products or others and assess their impact on the relative risk of mortality due to CVD in both adult men and women.

## Funding

The authors reported no funding received for this study.

## Author disclosures

The authors report no conflicts of interest.

## Data Availability

Data described in the manuscript, code book, and analytic code will be made available upon request, pending application and approval.
